# Chondroitin sulfate alleviates osteoporosis caused by calcium deficiency by regulating lipid metabolism

**DOI:** 10.1186/s12986-023-00726-3

**Published:** 2023-02-06

**Authors:** Tianshu Liu, Hai Yu, Shuai Wang, Huimin Li, Xinyiran Du, Xiaodong He

**Affiliations:** 1 Department of Epidemiology, School of Public Health, Cheeloo College of Medicine, Shandong University, Jinan, 250012 Shandong China; 2 Division of Cancer RNA Research, National Cancer Center Research Institute, Tokyo, 104-0045 Japan; 3 Department of Physical and Chemical Inspection, School of Public Health, Cheeloo College of Medicine, Shandong University, Jinan, 250012 Shandong China; 4 Institute for Medical Dataology, Shandong University, National Institute of Health Data Science of China, Jinan, 250012 Shandong China; 5 Institute of Toxicology, School of Public Health, Cheeloo College of Medicine, Shandong University, Jining, 250012 Shandong China; 6 College of Stomatology, Jining Medical University, Jining, 272067 Shandong China; 7 National Human Genetic Resources Center; National Research Institute for Health and Family Planning, Chinese Academy of Medical Sciences & Peking Union Medical College, Beijing, 100730 China

**Keywords:** Osteoporosis, Chondroitin sulfate, Intestinal flora, Metabolomics, Lipid metabolism

## Abstract

The use of non-drug intervention for calcium deficiency has attracted attention in recent years. Although calcium carbonate is the preferred raw material for calcium supplementation, there are few reports on the mechanism of the combined action of chondroitin sulfate and calcium to alleviate osteoporosis from the perspective of gut microbiota and metabolomics. In this study, a rat model of osteoporosis was established by feeding a low-calcium diet. The intestinal microbiota abundance, fecal and plasma metabolite expression levels of rats fed a basal diet, a low-calcium diet, a low-calcium diet plus calcium carbonate, and a low-calcium diet plus chondroitin sulfate were compared. The results showed that compared with the low calcium group, the calcium content and bone mineral density of femur were significantly increased in the calcium carbonate and chondroitin sulfate groups. 16 S rRNA sequencing and metabolomics analysis showed that chondroitin sulfate intervention could reduce short-chain fatty acid synthesis of intestinal flora, slow down inflammatory response, inhibit osteoclast differentiation, promote calcium absorption and antioxidant mechanism, and alleviate osteoporosis in low-calcium feeding rats. Correlation analysis showed that the selected intestinal flora was significantly correlated with metabolites enriched in feces and plasma. This study provides scientific evidence of the potential impact of chondroitin sulfate as a dietary supplement for patients with osteoporosis.

**Supplementary Information:**

The online version contains supplementary material available at 10.1186/s12986-023-00726-3.

## Introduction

In 1994, the World Health Organization proposed the concept of osteoporosis as a “progressive systemic skeletal disease characterized by low bone mass and microarchitectural deterioration of bone tissue, with a consequent increase in bone fragility and susceptibility to fracture” [1]. Osteoporosis in middle-aged and older adults is a global public health problem, especially in developing countries; therefore, it has attracted attention worldwide [2]. Decreased intestinal calcium absorption, insufficient dietary calcium intake, and chronic low-grade inflammation have been identified as risk factors for osteoporosis [3, 4]. Although the adverse sequelae of osteoporotic fractures are well documented and many effective treatment options are available [5], the treatment of osteoporosis often involves the use of medications, and adverse reactions associated with drug therapy have been a major challenge related to treatment goals. Therefore, the use of non-pharmacological interventions, including adequate nutrition and dietary supplements, rather than therapeutic strategies, for the treatment of osteoporosis has attracted much interest. To date, the focus has been on rapid bone loss in the postmenopausal period, which is mainly affected by estrogen deficiency, which masks the effects of calcium. The strong dependence of the effect of the calcium preparation used is often forgotten [6]. Research on HRT-free therapy has shown that calcium is the simplest and cheapest strategy to treat and prevent osteoporosis [7].

Calcium supplements are generally divided into two groups based on the nature of their chelating counterbalance ions with organic calcium supplements, including negatively charged organic molecules such as malate, citrate, fumarate and gluconate, and inorganic calcium supplements relying on inorganic chelating molecules such as carbonates, phosphates and chlorides [8]. Calcium carbonate and calcium citrate are the most common forms of calcium supplements [9]. Owing to its relatively high elemental calcium content (40%) and low price, calcium carbonate is recognized as the most cost-effective calcium source in China and abroad, and is the preferred raw material for calcium supplements [10]. Calcium supplementation may be an effective treatment for osteoporosis. However, calcium supplements alone are ineffective in preventing osteoporosis and can even increase the risk of fractures [11].

The combination of calcium supplements and vitamin D is considered the traditional strategy for osteoporosis treatment. But this method usually requires larger doses of calcium supplementation, which is less effective [12].

Chondroitin sulfates (CS) are a class of sulfated glycosaminoglycans, which are linear polysaccharides composed of repeated disaccharide units comprising uronic acid and N-acetylhexosamine [13]. In addition to being an anti-inflammatory molecule, CS also has antioxidant and lipid peroxidation inhibitory properties [14]. Animal studies have shown that chondroitin sulfate combined with probiotics can effectively inhibit the development of oxidative stress in experimental osteoarthritis rats and prevent the disorder of oxido-antioxidant balance [15]. In the United States, CS is approved as a dietary supplement for osteoarthritis, whereas in Europe and some other countries, it is used as a symptomatic slow-acting agent [16]. Previous studies have confirmed that the two sulfate groups of CS from different chains can interact with single calcium ions, induce the influx of calcium ions that may be involved in cell proliferation, and have a proliferative effect on chondrocytes, and the combination of CS and calcium is related to the calcification of cartilage [17]. However, there are very limited studies on the effects of the combined action of CS and calcium on osteoporosis.

Recently, the role of intestinal flora in metabolic diseases has been widely investigated. Several human diseases are linked to the dysregulation or imbalance in the microbial communities [18]. Evidence indicates that bone health may be regulated by the gut microbiome [19]. Intestinal flora is closely related to bone health. The existence of brain-bone, gut-bone, and brain-gut connections, referred to as the brain-gut-bone axis, may be one of the important regulatory factors affecting bone homeostasis through the regulation of its own metabolites, affecting host metabolism, transforming drug metabolism, and regulating intestinal barrier function [20, 21]. Adverse changes in the gut microbiome, such as dysbiosis, can lead to metabolic syndrome and inflammation, two important components of osteoarthritis progression [22]. Animal studies have also revealed that the gut microbiome regulates bone mass by altering the immune status and intestinal calcium uptake and influencing osteoclast-mediated bone resorption [23].

Metabolomics is a science that studies the changes in all metabolites in biological systems in response to external stimuli. Substrates and small molecule metabolites (< 1500 Da) of various metabolic pathways of products are assessed in specific physiological periods through qualitative and quantitative analysis and integration of information [24]. Plasma metabolomics characterizes all small molecule metabolites in the plasma, and fecal metabolites are closely related to intestinal microbiota activity [25]. However, the application of metabolomics in osteoporosis is just the beginning [26]. Based on the abundance of metabolites and cellular signaling molecules, particularly short-chain fatty acids, such as butyric acid which is produced by the gut microbiota, it is reasonable to consider that these imbalances may be related and should be investigated [27].

In this study, using fecal and plasma metabolomics to assess gut microbes, we compared the outcomes of CS and calcium carbonate intervention in an osteoporotic rat model. Our aim was to better understand the role of CS in improving osteoporosis and its underlying mechanisms, which may provide a new approach for the remission of osteoporosis caused by calcium deficiency.

## Material and methods

### Chondroitin sulfate reagent

Chondroitin sulfate was purchased from ORIHIRO LTD., Japan. Samples are white tablets. The recommended dose for humans is 4.2 g/60 kg. Each 2.5 g contains 17 mg of calcium and 10 mg of magnesium.

### Animal experiment and design

Weaned female Wistar rats weighing 60–75 g, and of SPF grade at approximately four weeks of birth were selected. The rats were provided by Jinan Pengyue Experimental Animal Breeding Co., Ltd under experimental animal production license no. SCXK (Lu) 20190003 and experimental animal use license no. SYXK (Lu) 20150015. The rats were house in an environment with an ambient temperature of 22 ± 2 °C and relative humidity at 50% ± 10%. After adaptive feeding, rats were fasted for 12 h, weighed, and randomly divided into normal control group, low calcium control group, calcium carbonate control group, and Chondroitin sulfate group according to body weight, with 10 rats in each group. The normal group was fed a basal diet and deionized water, and the other groups were fed a low-calcium basal diet and deionized water. Oral intragastric was administered at 1 mL/100 g bw. The recommended intake of Chondroitin sulfate is 4200 mg per person per day, based on 60 kg weight per person, equivalent to 70 mg/kg.bw per day. The oral dose of Chondroitin sulfate was determined to be 30 times the recommended dose of human body, that is, the daily dosage of rats in each group was 2100 mg/kg.bw. In the calcium carbonate group, calcium carbonate was 525 mg/kg body weight (40% calcium content), and the concentration of calcium carbonate solution was 52.5 mg/mL. The normal control group and the low calcium control group were gavaged with the same volume of deionized water instead of the test solution. Bone mineral density (BMD) function was tested after three months of continuous administration. This experiment was approved by Shandong University Preventive Medicine Animal Experiment Ethics Committee, and the study’s protocols adhere to the ethics and welfare requirements of the National Institutes of Health (NIH).

The low calcium base feed formula is listed in Table 1.

**Table 1:** Low calcium base feed formula (Ca2 + meter, 150 mg/100 g feed)

Composition	%	Composition	%
Casein	10.0	Salt-mixture^b^	2.6
Soybean meal^a^	15.0	Mixed vitamins^c^	1.0
Wheat flour	54.0	Choline chloride	0.2
Corn oil	4.0	Dl – methionine	0.2
Cellulose	2.0	Starch	11.0

^a^Need to be used after high pressure treatment

^b^Each component content per kg of salt-mixture is: KH_2_PO_4_ 501.4 g; NaCl 74.0 g; MgCO_3_ 50.2 g; Ferrous lactate 5.4 g; Lactic acid zinc 4.16 g; MnCO_3_ 3.5 g; CuSO_4_·5H_2_O 0.605 g; Na_2_SeO_3_ 6.6 mg; KI 7.76 mg; Cr_3_Cl·6H_2_O 0.292 g; add sucrose to 1 kg

^c^Each component content per kg of mixed vitamins is: vitamin A 400,000 IU; vitamin D_3_ 100,000 IU; vitamin E 500 IU; vitamin K 5 mg; vitamin B_1_ 600 mg; vitamin B_2_ 600 mg; vitamin B_6_ 700 mg; vitamin B_12_ 1 mg; Nick acid 3000 mg; Folic acid 200 mg; Calcium pantothenate 1.6 g; biotin 20 mg, add sucrose to 1 kg

#### Femur gravimetric determination

The rats were euthanized after three months. The right femur was removed and dried in an oven at 105 °C until constant weight was obtained. The backbone was also weighed.

#### Bone densitometry

The right femur was dried to constant weight, and BMD was measured at the midpoint of femur and distal end of femur using the xR-600 dual-energy X-ray BMD instrument (NORLAND).

#### Determination of bone calcium

The right femur was dried to constant weight, placed in a crucible, charred in an electric furnace, and ash combusted in a muffle furnace at 550 °C. The calcium content of femur was determined by EDTA titration.

#### DNA extraction and 16S rRNA amplification and sequencing

The genomic DNA of the samples was extracted using the cetyltrimethylammonium bromide (CTAB) method, and the purity and concentration of DNA were tested by agarose gel electrophoresis. The DNA sample was subsequently diluted to 1 ng/μL with sterile water in a centrifugal tube. The 16S rRNA genes in different regions were amplified using a barcode-specific primer. The same volume of IX loading buffer (containing SYB green) was mixed with polymerase chain reaction (PCR) products. PCR products were detected by electrophoresis with 2% agarose gel. The qualified PCR products were purified using magnetic beads, quantified by enzyme standard, and mixed in equal quantities according to the concentration of PCR products. After full mixing, the PCR products were detected by 2% agarose gel electrophoresis, and the target bands were recovered using gel recovery kits provided by QIAGEN. TruSeq® DNA PCR-free Sample Preparation Kit was used for library construction. The constructed library was quantified by Qubit and Q-PCR. The samples were then sent for sequencing using NovaSeq6000.

#### Bioinformatics analysis of intestinal microbiota

Quality control was carried out in accordance with QIIME version 1.9.1 [28] to obtain high-quality clean tags [29]; subsequently, raw tags were quality filtered. Chimera sequences were detected and removed [30] using the UCHIME algorithm [31]. Uparse software version 7.0.1001 was used for operational taxonomic unit (OUT) clustering with SILVA database [32] performed at 97% sequence similarity. Alpha and beta diversity estimations were then performed using QIIME version 1.7.0. Lefse analysis was used to compare the differential metabolites of the four groups, and differential metabolites were sequentially compared between the normal control group (group N) and low calcium control group (group C) and between low calcium control (group C) and Chondroitin sulfate group (group CS) and between calcium carbonate control group (group Ca) and Chondroitin sulfate group(group CS). Relative intestinal microbiota abundance at phylum level in four groups were accessed using prism8.

#### Fecal metabolomics and plasma metabolomics

Target-like metabolomics is based on a SCIEX QTRAP® 6500 + mass spectrometer with triple quadrupole-linear ion trap and Multiple Reaction Monitoring (MRM) to accurately characterize and quantify metabolites in biological samples. The experimental process mainly includes sample collection, metabolite extraction, mass spectrometry detection and data analysis. Qualitative analysis of parent ion (Q1), daughter ion (Q3), retention time (RT), cluster potential (DP) and collision energy (CE) was carried out. The SCIEX OS version 1.4 was used to open the mass spectrum file, and the chromatographic peak integration and correction work was performed. The metabolites were interpreted by KEGG (http://www.genome.jp/kegg/), HMDB (http://www.hmdb.ca/), and Lipidmaps database (http://www.lipid maps.org/). Quality control through the QC sample overlap diagram and the correlation analysis were performed. After data conversion using metaX [33], principal component analysis (PCA) was conducted. The statistical significance of each metabolite between the normal control and low calcium control group, and low calcium control group and Chondroitin sulfate group, and calcium carbonate control group and Chondroitin sulfate group were calculated using Student's unpaired t-test. Subsequently, multiple difference of metabolites, namely foldchange (FC) value between the normal control and low calcium control group, and low calcium control group and Chondroitin sulfate group, and calcium carbonate control group and Chondroitin sulfate group were calculated. The default criteria for screening differential metabolites were VIP > 1, *P* < 0.05 and FC > 1.2 or FC < 0.833. Ipath was used to analyze whether the intestinal microbiota were associated with metabolic pathways. The KEGG database was used to analyze the function and metabolic pathway of metabolites between the normal control and low calcium control group, and low calcium control group and Chondroitin sulfate group, and calcium carbonate control group and Chondroitin sulfate group. All graphs and plots were generated using ggplot2 package in R.Novogene provides 16S RNA sequencing and metabolomics services.

#### Statistical analysis

P-value and rho were calculated between intestinal microbiota and fecal metabolites using Spearman’s statistical method. A *P*-value of < 0.05 and absolute value of rho ≥ 0.8 were considered statistically significant.

## Results

### Effects of calcium carbonate and CS intervention on physiological parameters in low-calcium diet fed rats

**Fig. 1:**
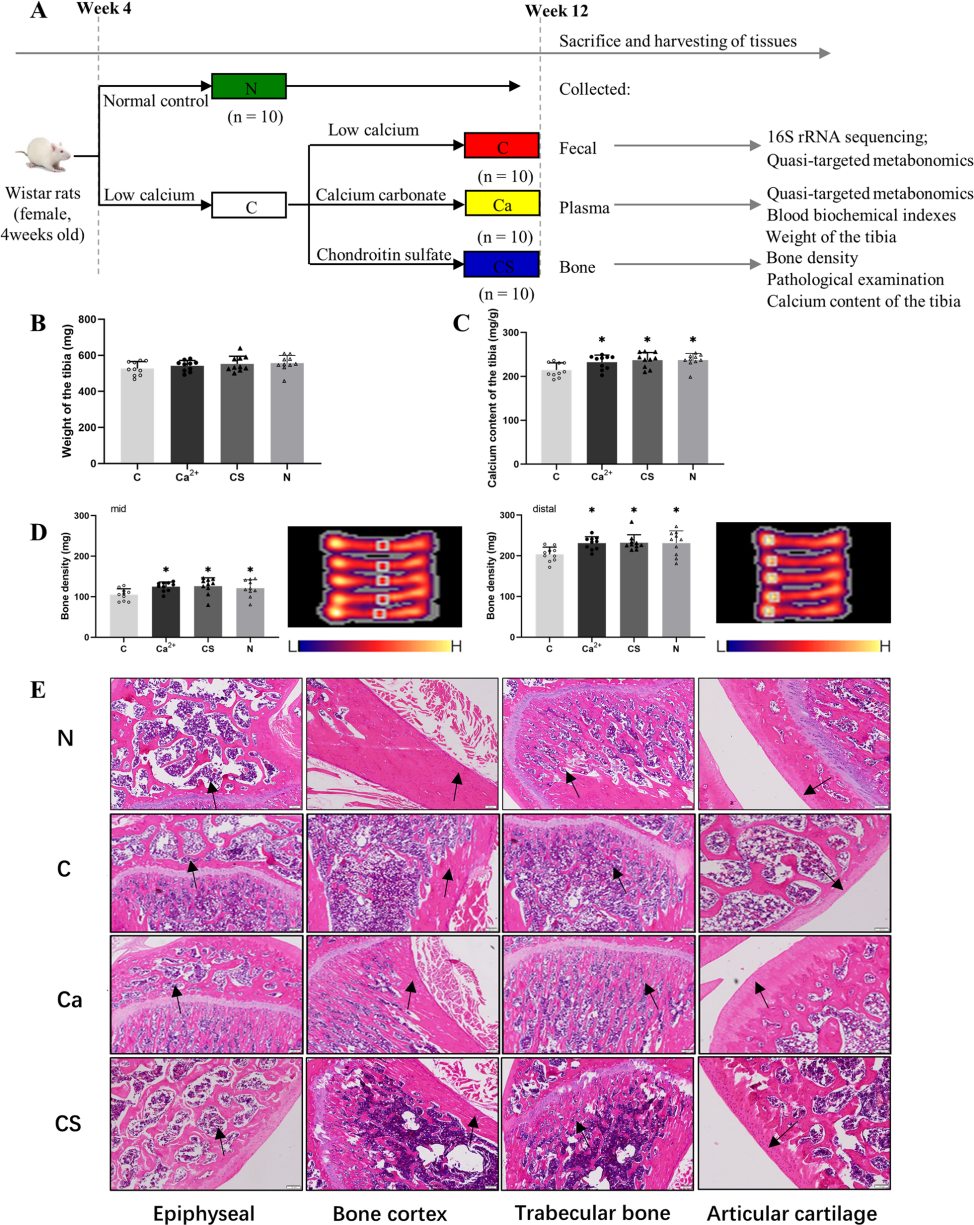
Animal experiment plan and effects of various interventions on the femur of the rats. **A** Animal experiment plan. The changes of femoral shaft weight (**B**), bone calcium content (**C**), and bone mineral density (**D**) of the femurs from the rats subjected to the various interventions. HE staining showed bone mineral density of rats (as shown by arrow) in different groups (**E**). All data is represented as Mean ± Standard Deviation. All data were accessed using One-way ANOVA with Tukey’s multiple comparisons test. *P < 0.05 indicate the significant difference

The schematic diagram of the animal experiment is shown in Fig. 1A and the low-calcium base feed formula is shown in Table 1. After 12 weeks of intervention, femoral shaft weight, bone calcium content, and bone mineral density (BMD) were evaluated in the three groups (Fig. 1B–D). There was no significant difference in femoral shaft weight between low calcium group (group C) and normal control group (group N), cartilage sulfate group (group CS) and calcium carbonate group (group Ca) (*P* > 0.05). On the other hand, The calcium content of femur, bone mineral density at the midpoint of femur and distal femur in group C were significantly lower than those in groups N, Ca and CS (0.05 and 0.01, respectively). (*P* < 0.05, *P* < 0.01). The calcium content of femur, bone mineral density of midpoint of femur and bone mineral density of distal femur in CS group were not lower than those in Ca group. HE staining showed that compared with group C, bone mineral density was significantly increased in group CS and Ca (Fig. 1E).

### Structural changes in intestinal microflora induced by calcium carbonate and CS intervention

**Fig. 2:**
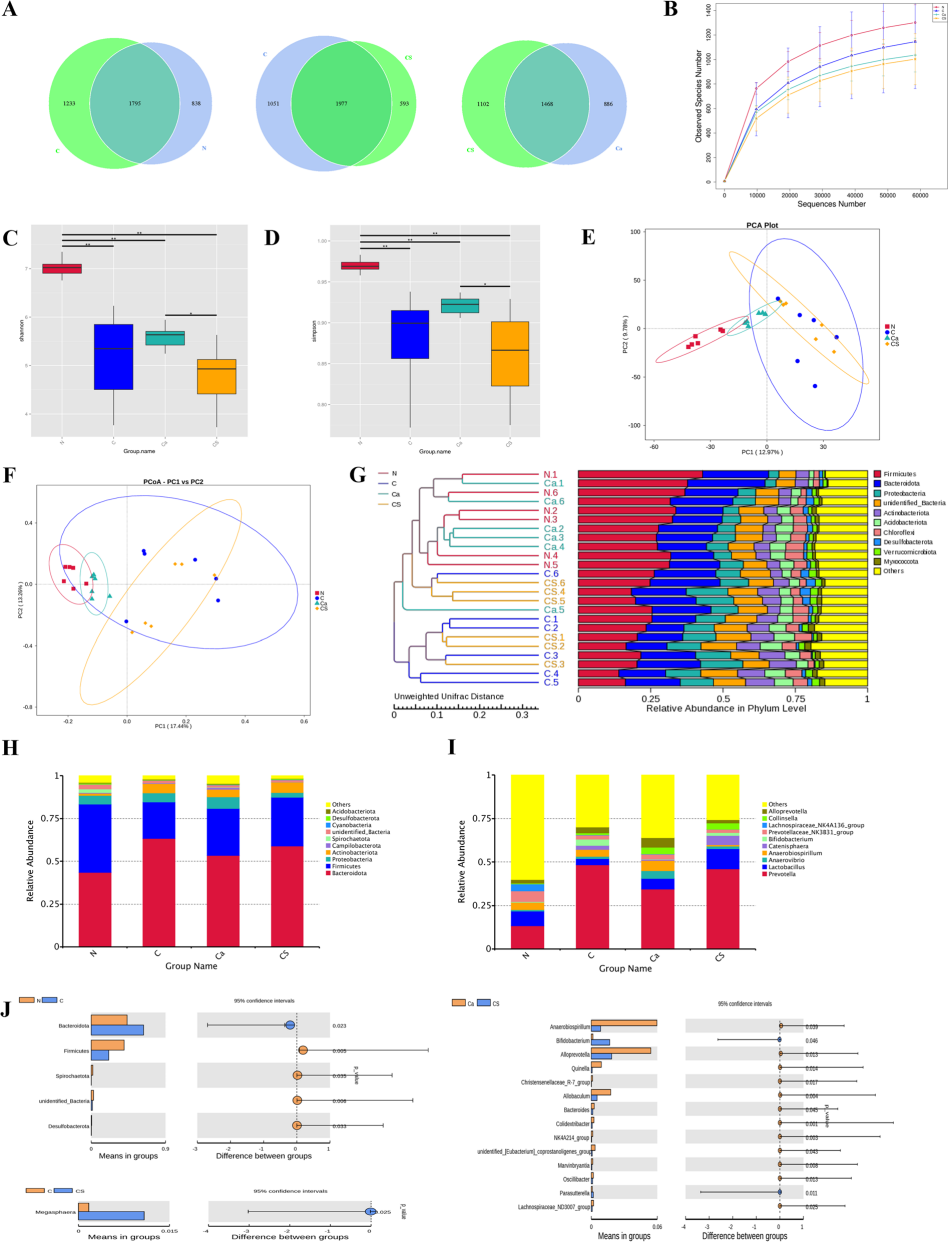
Taxonomic Composition of Intestinal Microbiota. **A** Venn diagram of C and N groups and C and CS groups and CS and Ca groups at OTU level. Individual sparse curves (**B**), Shannon index (**C**), Simpson index (**D**). **E** PCA Plot. **F** PCoA Plot. The abscissa represents the first principal component (PC1), the ordinate represents the second principal component (PC2). **F** Weighted UniFrac PCoA diagram. **G** Cluster analysis of intestinal microbiota between samples. **H** Composition of intestinal microbiota at phylum level. (I) Composition of intestinal microbiota at genus level. **J** at the genus level (*P* < 0.05, or P < 0.01) different gut microbiota

A total of 3866 operational taxonomic units (OTUs) were found in the N group and the C group, with 838 and 1233 OTUs specific to the two groups, respectively (Fig. 2A). Individual sparse curves approached saturation platforms, indicating high sampling coverage (over 99%) in all samples (Fig. 2B). The Shannon and Simpson index of α diversity was significantly different in the N group and the C groups (Fig. 2C, D). There were 3621 OTUs in the C group and the CS group, with 1051 and 593 OTUs specific to the two groups, respectively (Fig. 2A). The Shannon and Simpson index did not differ between the two groups (Fig. 2C, D). There were 3456 OTUs in the CS and C groups, with 1102 OTUs in the CS group and 886 OTUs in the C group (Fig. 2A). There were significant differences in the Shannon and Simpson indexes in the two groups (Fig. 2C, D). This suggests that calcium has a stronger ability to reduce intestinal flora richness and α diversity under CS intervention.

In terms of β diversity, the similarity of the bacterial communities in the three groups is shown in Fig. 2. Group N was distinguished from group C and group N from group CS according to the weighted UniFrac PCA score (Fig. 2E). Meanwhile, the weighted UniFrac PCoA score distinguished the CS group from the N group (Fig. 2F). An unweighted pair group method with arithmetic mean and clustering tree of weighted UniFrac was used to cluster the differences between the samples (Fig. 2G). The multiple response permutation procedure analysis showed significant differences in microbial community structure between the N group and the C group, and between the CS group and the Ca group (Additional file 1: Table S1). Therefore, calcium has a strong ability to affect the diversity of intestinal microbiota under CS intervention but cannot completely restore it.

Figure 2H, I show the composition of intestinal flora. The top 10 phyla and genera in the most abundant microbiome of all participants are shown in Fig. 2H, I, and included Bacteroidota, Firmicutes, Proteobacteria, Actinobacteriota, Campilobacterota, Spirochaetota, unidentified_Bacteria, Cyanobacteria, Desulfobacterota, Acidobacteriota, which were dominant in all microbial communities. Compared with the N group, the C group showed reduced Firmicutes, Spirochaetota, unidentified_Bacteria, and Desulfobacterota and increased Bacteroidota. CS intervention resulted in significant changes in intestinal microbial community structure. Specifically, Megasphaera was increased with CS intervention compared with the C group. CS intervention elevated Bifidobacterium levels to be higher than the Ca group, and Anaerobiospirillum, Alloprevotella, Quinella, Christensenellaceae_R-7_group, Allobaculum, Bacteroides, Colidextribacter, NK4A214_group,unidentified_[Eubacterium]_coprostanoligenes_group, Marvinbryantia, Oscillibacter, Parasutterella, and Lachnospiraceae_ND3007_group were all lower than the Ca group (Fig. 2J).

### Effects of low-calcium diet on intestinal microbiota and metabolomics

**Fig. 3:**
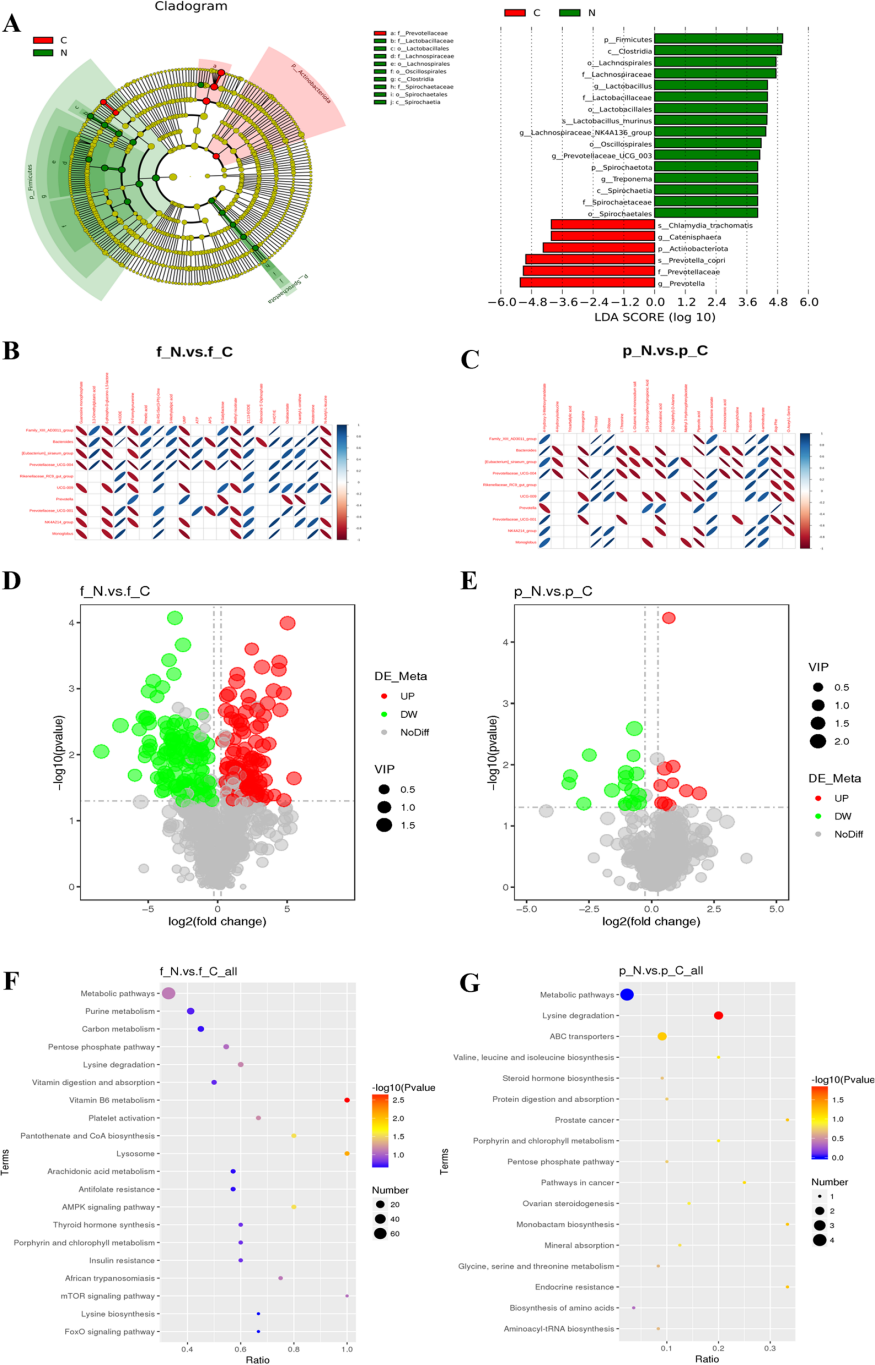
Effect of low calcium diet on intestinal microbiota and metabolic profiles. **A** LEfSe analysis of cladogram in group N and group C. **B** The correlation graph shows the correlation between intestinal microbiota with fecal metabolites. **C** The correlation graph shows the correlation between intestinal microbiota with plasma metabolites. **D** The volcano map of fecal metabolites between the N group and C group. **E** The volcano map of plasma metabolites between the N group and C group. **F** The KEGG bubble map of fecal metabonomics between the low calcium group and the control group. **G** The KEGG bubble map of plasma metabolites between the low calcium group and the control group. The larger the Abscissa in the picture, the higher the enrichment of differential metabolites in the pathway. The color of the point represents the P-value value of the hypergeometric test, and the smaller the value is, the greater the reliability of the test is and the more statistically significant it is. The size of the point represents the number of differential metabolites in the corresponding pathway, and the larger the point, the more differential metabolites in the pathway

A linear discriminant analysis effect size (LEfSe) analysis was used to identify potential biomarkers for intestinal flora in the N group and the C group, and a t-test was used to further examine and visualize the results (*P* < 0.05; Fig. 3A). A total of 22 significant taxa were identified. There were 16 potential biomarkers of intestinal flora in the N group and eight in the C group. On this basis, we analyzed the correlation between 10 intestinal flora biomarkers and 20 fecal and plasma metabolites involved in the KEGG pathway in these two groups. The results showed that there was a strong correlation between intestinal flora and fecal and plasma metabolic pathways (Fig. 3B, C). To assess the global metabolic changes in the C group, fecal metabolites from N and C rats were measured and analyzed by mass spectrometry combined with liquid chromatography (LC–MS/MS). The fecal metabolites in the N group increased by 106 species and decreased by 117 species (Fig. 3D). Figure 3F shows the KEGG bubble diagram of the metabolic pathways involved in the production of different metabolites. Compared with the C group, metabolites in the N group were significantly different, including nucleosides, nucleotides and analogues, organic oxygen compounds, heterocyclic compounds, and organic oxygen compounds. These differentially expressed metabolites were enriched in the KEGG pathway, and they involve vitamin B6 metabolism, lysosome, pantothenic acid and CoA biosynthesis, and the AMPK signaling pathway (Additional file 1: Table S2). These pathways may be assigned to cofactor and vitamin metabolism, transport and catabolism, and signal transduction. Abundances of the 10 kinds of plasma metabolites were higher in the N group than those in the C group, and 18 kinds of plasma metabolites were lower in the N group than those in the C group (Fig. 3E). Figure 3G shows the KEGG bubble diagram. The differentially expressed metabolites in plasma were enriched in the KEGG pathway and allocated to the lysine degradation of amino acid metabolism (Additional file 1: Table S3).

### Effects of calcium carbonate on intestinal microflora and metabolomics

**Fig. 4:**
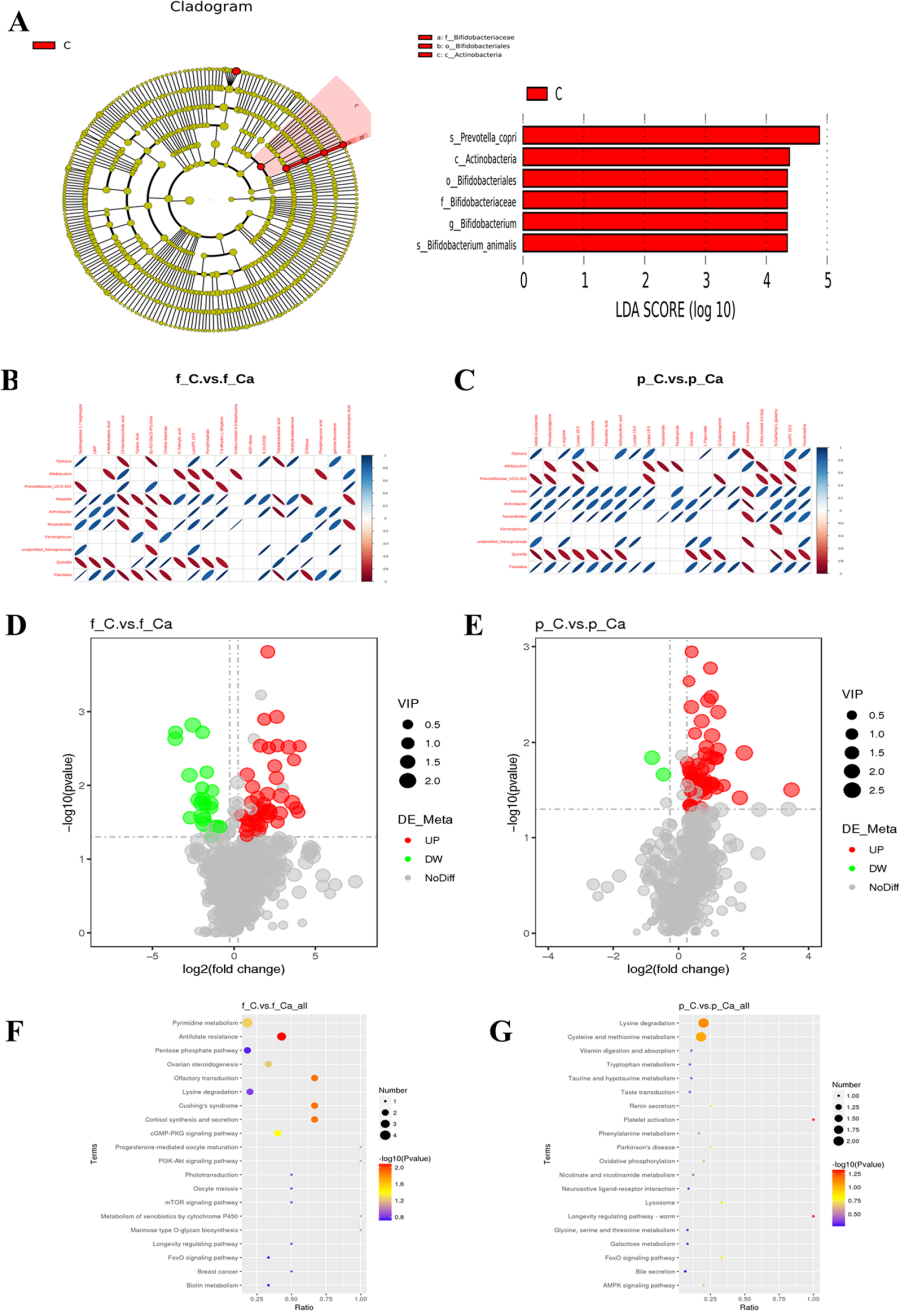
Calcium carbonate improved intestinal microbiota and metabolic disorders induced by low calcium diet. **A** LEfSe analysis of cladogram in C group and Ca group with LDA Score larger than 3. **B** The correlation graph shows the correlation between intestinal microbiota and fecal metabolites in group C and group Ca. **C** Correlation graph shows the correlation between intestinal microbiota and plasma metabolites in group C and group Ca. **D** The volcano map of fecal metabolites between the C group and Ca group. **E** The volcano map of plasma metabolites between the C group and Ca group. **F** The KEGG bubble map of fecal metabonomics between the C group and Ca group. **G** The KEGG bubble map of plasma metabolites between the C group and Ca group. All data is represented as Mean ± Standard Deviation

Through 16S rRNA sequencing and metabonomics analysis, we compared potential biomarkers belonging to intestinal flora with differential metabolites in feces and plasma of groups C and Ca to elucidate the mechanism of calcium carbonate action. A total of 6 taxa were identified as significant (Fig. 4A), with Prevotella copri and Actinobacteria significantly present in group C. Concurrently, we analyzed the correlation between 10 intestinal flora biomarkers and 20 fecal and plasma metabolites involved in the KEGG pathway in groups C and Ca. The results showed that there was a strong correlation between intestinal flora and fecal and plasma metabolic pathways (Fig. 4B, C). To assess the global metabolic changes in the Ca group, fecal metabolites from the C and Ca groups were measured and analyzed by LC–MS/MS. The fecal metabolites in group C increased by 42 and decreased by 22 (Fig. 4D). Figure 4E shows the metabolic pathways involved in the production of different metabolites. Compared with Ca group, the changes in the metabolites in group C included up-regulation of AMP, GMP, PRPP and down-regulation of progesterone and 17 alpha-hydroxyprogesterone. These differentially expressed metabolites are enriched in KEGG pathways, including Antifolate resistance, olfactory transduction, cortisol synthesis and secretion, Cushing's syndrome, and the cGMP-PKG signaling pathway (Additional file 1: Table S2). These pathways may be assigned to antitumor, sensory, endocrine, and signal transduction. Group C had 52 more plasma metabolites than group Ca, and 2 plasma metabolites fewer than group Ca (Fig. 4F). Figure 4G shows the KEGG bubble diagram. Calcium carbonate intervention down-regulated plasma nicotinamide, which is involved in the longevity regulating pathways—worm and platelet-activated ADP (Additional file 1: Table S3).

### Effects of chondroitin sulfate on intestinal microflora and metabolomics

**Fig. 5:**
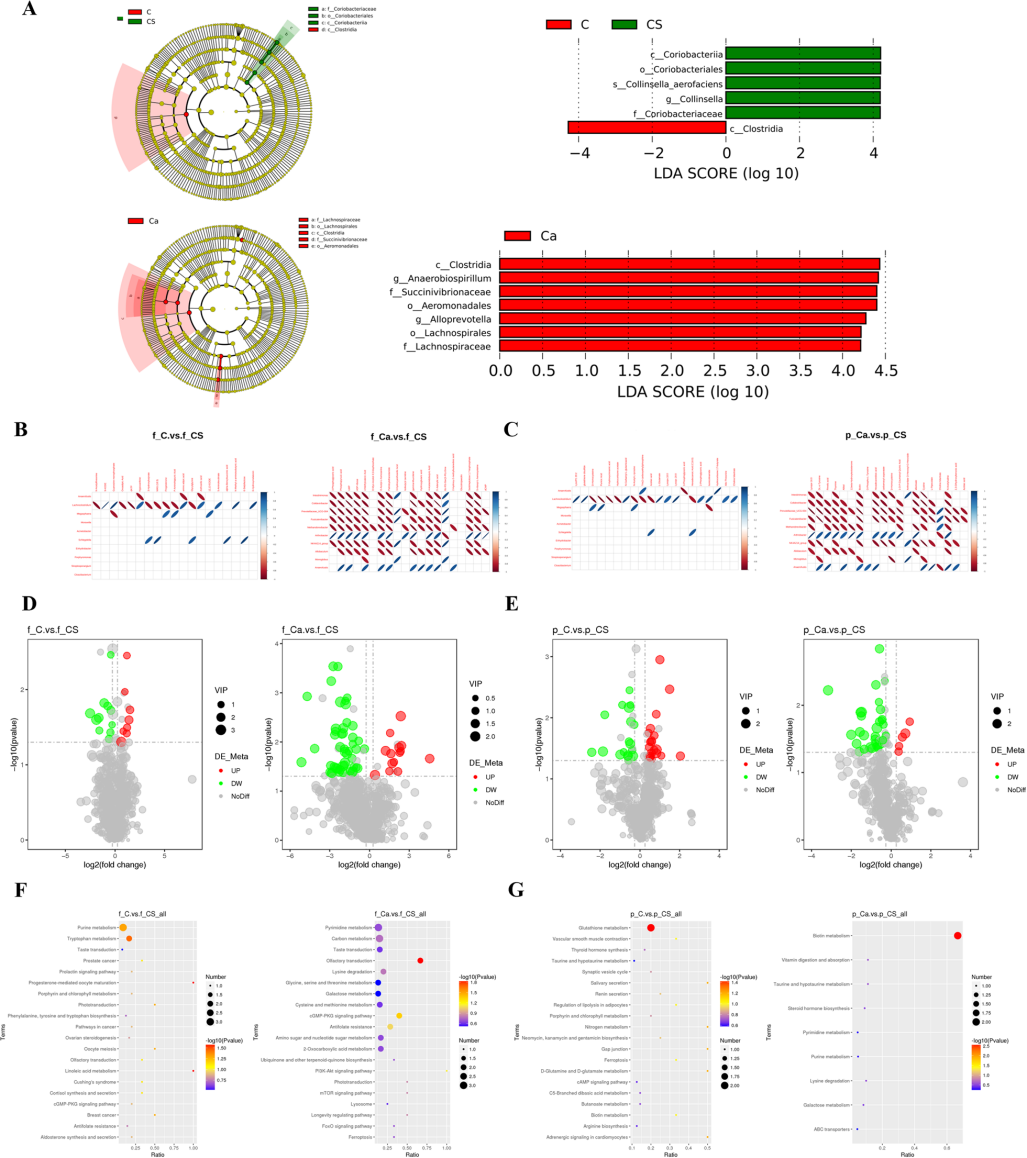
Calcium carbonate improved intestinal microbiota and metabolic disorders induced by low calcium diet. **A** LEfSe analysis of cladogram in C and CS group and CS and Ca group with LDA Score larger than 3. **B** The correlation graph shows the correlation between intestinal microbiota with fecal metabolites. **C** The correlation graph shows the correlation between intestinal microbiota with plasma metabolites. **D** The volcano map of fecal metabolites between the C and CS group and CS and Ca group. **E** The volcano map of plasma metabolites between the C and CS group and CS and Ca group. **F** The KEGG bubble map of fecal metabonomics between the C and CS group and CS and Ca group. **G** The KEGG bubble map of plasma metabolites between the C and CS group and CS and Ca group. All data is represented as Mean ± Standard Deviation

LEfSe was used to identify biomarkers characterizing dimensional intestinal bacteria and to identify taxonomic groups with significant differences between groups, and to generate a clade map. A total of six and seven taxa were identified as significant in the comparison of the C group and the CS group (Fig. 5A). Specifically, in the comparison between these two groups where the C group showed Clostridia, the number of Coriobacteriaceae and Collinsella and Collinsella_aerofaciens and Coriobacteriales and Coriobacteriia was higher in the CS group. In the comparison between the C group and CS, numbers of Lachnospiraceae, Lachnospirales, Alloprevotella, Aeromonadales, Succinivibrionaceae, Anaerobiospirillum, and Clostridia were higher in the latter group. Concurrently, we analyzed the correlation between 10 intestinal flora biomarkers and 20 fecal and plasma metabolites involved in the KEGG pathway in the C group and CS group. The results showed that there was a strong correlation between intestinal flora and fecal and plasma metabolic pathways (Fig. 5B, C). To assess the global metabolic changes in the CS group, fecal metabolites and plasma metabolites from the C and CS groups were measured and analyzed by LC–MS/MS. Compared with the C group, fecal metabolites in the CS group increased by 11 and decreased by 8 (Fig. 5D). The differentially expressed metabolites were enriched in linoleic acid metabolism, progemediated oocyte maturation, tryptophan metabolism, and purine metabolism in the KEGG pathway (Additional file 1: Table S2). Figure 5F shows the metabolic pathways involved in the production of different metabolites. These pathways may be assigned to lipid metabolism, the endocrine system, amino acid metabolism and nucleotide metabolism. The plasma metabolites in the CS group were 18 more than those in the C group and 21 less than those in the Ca group (Fig. 5E). Figure 5G shows the KEGG bubble diagram. Plasma differentially expressed metabolites are enriched in the KEGG pathway and assigned to the glutathione metabolism of other amino acids (Additional file 1: Table S3). These fecal metabolites and plasma metabolites have a variety of structures, including lipids, fatty acids, and amino acid derivatives.

**Fig. 6:**
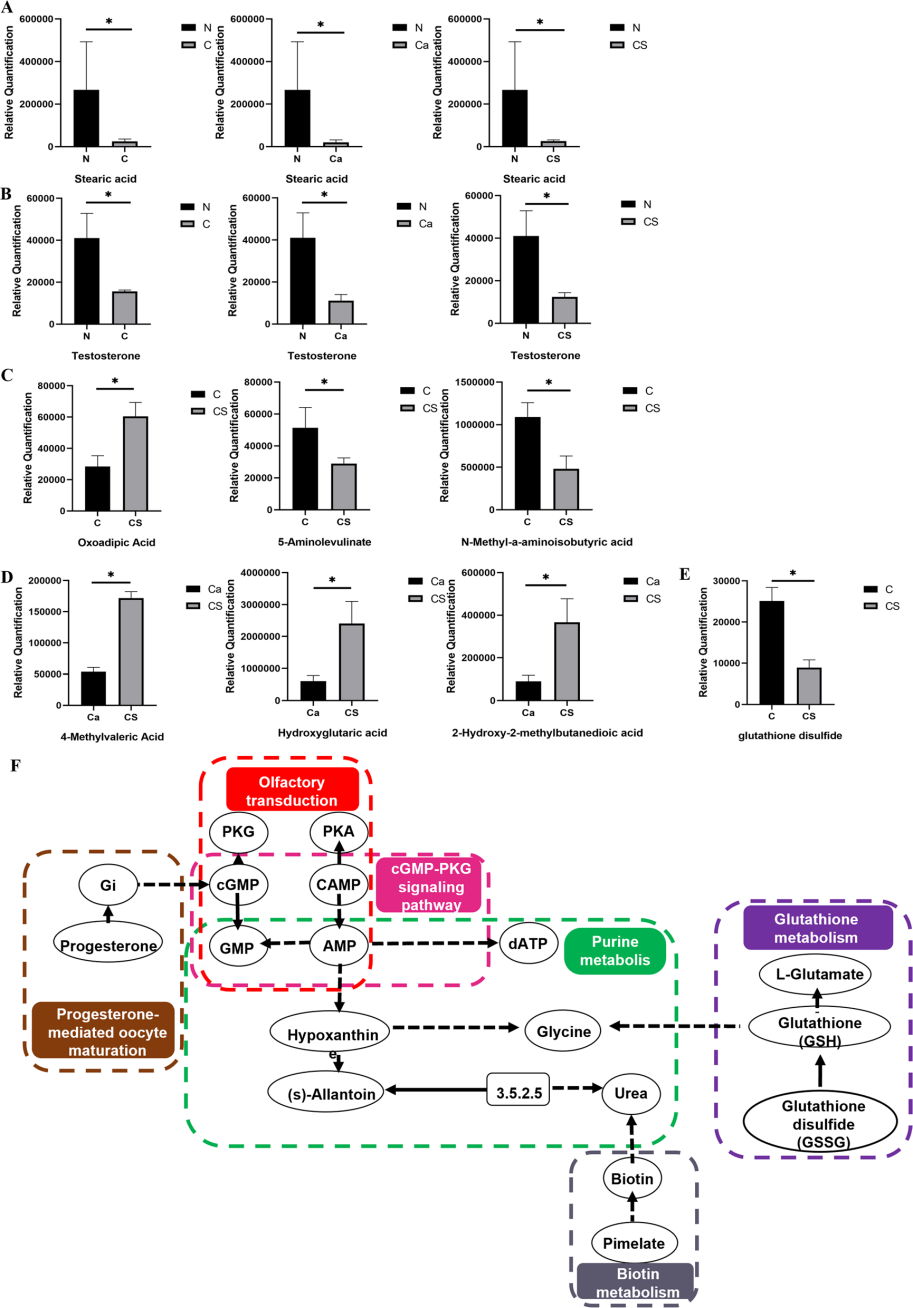
Effect of chondroitin sulfate intervention on excreta and plasma metabolites in rats. **A** Differences in fecal stearic acid between normal and different intervention groups. **B** Differences in plasma testosterone between normal and different intervention groups. **C**, **D** Fecal short-chain fatty acid levels in different intervention groups. **E** Difference level of glutathione between chondroitin sulfate and low calcium group. **F** The metabolic pathways affected by chondroitin sulfate intervention

Compared with the Ca group, the number of fecal metabolites in the CS group increased by 49 and decreased by 14 (Fig. 5D). Differentially expressed metabolites are enriched in olfactory transduction and the CGMP-PKG signaling pathway in the KEGG pathway (Additional file 1: Table S2). Figure 5F shows the metabolic pathways involved in the production of different metabolites. These pathways may be assigned to sensory system functions and signal transduction. The plasma metabolites in the CS group were 33 more than those in the Ca group and 5 less than those in Ca group (Fig. 5E). Figure 5G shows the KEGG bubble diagram. The differentially expressed metabolites in plasma were enriched in the KEGG pathway and allocated to biotin metabolism of cofactors and nutrition (Additional file 1: Table S3). These fecal and plasma metabolites have a variety of structures, including lipids, fatty acids, and amino acid derivatives. The metabolic pathways affected by CS intervention are shown in Fig. 6F.

We detected 843 compounds from 2200 + compound controls based on the Novo self-established metabolome database. In the analysis of differential metabolites in feces and plasma, the number of metabolites identified in feces was much greater than that in plasma. Among 290 + lipids (100 + oxidized lipids, 190 + lipids), 107 lipids were detected, among which 93 lipids were different, we obtained lipid classification by matching screened DEL with Lipidmaps database (http://www.lipidmaps.org), delete mismatched entries and count the number of DEL that accompany each category, the most highly ranked category is Fatty Acyls (FA). The levels of plasma testosterone and stearic content in normal feeding rats were significantly higher than those in the other two groups (Fig. 6A, B), indicating that the plasma testosterone and stearic content in the feces of normal feeding rats were decreased due to long-term low calcium levels, and the supplementation of calcium and CS could not be recovered. In the fatty acids metabolites, we confirmed differential changes in short-chain fatty acids in feces, but not in plasma. CS intervention resulted in higher levels of absolute oxoadipic acid and lower levels of absolute 5-aminolevulinate and N-methyl-a-aminoisobutyric acid content in the Ca group compared with the C group (Fig. 6C), and lower levels of absolute 4-methylvaleric acid and hydroxyglutaric acid and 2-hydroxy-2-methylbutanedioic acid content in the C group compared with the Ca group (Fig. 6D). However, in plasma metabolomics, we found that CS induced antioxidant activity and reduced the oxidized glutathione content (Fig. 6E).

## Discussion

In this study, we used 16 s rRNA sequencing and analyses of fecal and plasma metabolites to evaluate intestinal flora from the feces of an osteoporotic rat model. We found that CS and calcium carbonate administration not only alleviated osteoporosis, but also affected the composition of gut microbes and metabolic features. Some of the altered metabolites were functionally correlated with the disturbance of intestinal flora. In this study, CS inhibited oxidative stress in rats with osteoporosis. CS may be a promising new non-drug treatment for osteoporosis.

Our results are the first to demonstrate that a low-calcium diet may cause significant changes in the proportion and abundance of gut microbiome. Moreover, CS can partially restore intestinal disorders. Firmicutes (F) and Bacteroidetes (B) were the dominant bacteria in the three groups in this study. Previous studies have also suggested that changes in gut microbiota composition are involved in the development of osteoporosis [34].

In this study, a low-calcium diet significantly reduced Firmicutes and Lactobacilli, and increased Actinobacteriota and Prevotella at the phylum and genus levels. Firmicutes are positively correlated with calcium absorption [35]. They activate osteoclasts and increase inflammation [36]. Moreover, we found that Lactobacilli belonging to Firmicutes, which are commonly used as probiotics, are associated with calcium deficiency. Increased Lactobacillus abundance has been reported to prevent bone loss [37], possibly due to its anti-obesity and anti-inflammatory activities [38]. Interestingly, studies have found a positive correlation between bone mineral density and members of the Actinomycota group (which includes the Bifidobacteria family) [39]. A reduction in the Actinobacteriota phylum members may lead to the translocation of lipopolysaccharides into serum, which then leads to a reduction in bone mass through inflammatory pathways [40]. This is consistent with our research, which showed that the Prevotella spp. abundance was increased by a low-calcium diet and partially restored by calcium carbonate. The Prevotella genus is associated with inflammatory bone loss and is rapidly increased and maintained at high abundance in the intestines of osteoporotic rats [41]. A higher risk of osteoporosis has been shown to be associated with higher levels of Clostridium [42] and lower levels of Collinsella [43] spp. in the intestinal flora of low-calcium rats, induced by CS intervention. The overproduction of lipopolysaccharide (LPS) by intestinal microorganisms significantly increases the production of inflammatory factors tumor necrosis factor -α (TNF-α) and interleukin (IL)-1β, triggering an inflammatory response that leads to bone mineral loss [44]. Compared with calcium supplementation alone, CS intervention significantly upregulated intestinal bifidobacteria, participated in intestinal microbiota homeostasis, reduced intestinal barrier and LPS, and produced SCFA, which regulate inflammation through the activation of free fatty acid 2 and free fatty acid 3 receptors and GPR109A or inhibition of histone deacetylases. GPR109A in the metabolite-sensing G-protein coupled receptors (GPCR) family can inhibit the expression and secretion of TNFα, IL-6 and monocyte chemoattractant protein-1 induced by Toll-like receptor (TLR)4 [45]. TLR4 is directly involved in the expression of osteoblasts, osteoclasts, and mesenchymal stem cells in addition to directly regulating the inflammatory response in the bone [46]. In addition, SCFAs also reduce intestinal pH. Acidic pH stimulates T cell death-associated gene 8 in the proton-sensing GPCR family, leading to decreased IL-6 and TNFα production after LPS stimulation in peritoneal macrophages through a cAMP-dependent mechanism [47], is involved in the anti-inflammatory reaction, and increases mineral absorption [48]. Overproduction of LPS by the gut microbiome may lead to bone mineral loss through inflammatory pathways; however, the involvement of Bifidobacterium in the homeostasis of the gut microbiome, intestinal barrier, and reduction of LPS further inhibits osteoporosis through anti-inflammatory mechanisms. According to previous studies, as a human therapeutic agent, CS alone has low bioavailability, which is only about 0–13% of the total oral intake, and most strains capable of degrading CSA are classified as Bacteroidetes [49]. A number of previous studies have found that the relative abundance of Bacteroides is positively correlated with osteoporosis. In our study, CS reduced Bacteroides, which is consistent with previous study results [43, 44]. Lachnospiraceae is one of the biomarkers of osteoporosis in the intestinal flora. Trichospiraceae can decompose complex polysaccharides into SCFAs [50], which mediate changes in the insulin-like growth factor-1 level caused by microbiota and contribute to the effect of colonization on bone conversion [44]. The abundance of both Bacteroidetes and Trichospiraceae may play a positive role in bone metabolism and fracture risk. In fact, although some bacteria, including anaerobic Spirularia, have the potential to become biomarkers, these bacteria have been poorly studied, and the specific mechanism between the occurrence and development of osteoporosis is still unclear. Further functional investigation is needed to verify this hypothesis.

Previous studies have analyzed changes in microbial composition during calcium supplementation, dietary calcium acted in a prebiotic manner by promoting significant increases in two species analyzed from Bacteroidetes and Actinobacteria phyla (Bacteroides/Prevotella and Bifidobacterium) [51]. In addition, CS—treated mice were found to increase the abundance of Lactobacillus and Bacteroides [52, 53]. In our study, low calcium led to an increase in actinobacteria and Prevotela and a decrease in lactobacillus. We mitigated the effects of low calcium by feeding calcium carbonate and chondroitin sulfate, but CS supplementation can be used as an exogenous substrate for Bacteroides [53]. Calcium provides sufficient environment for the growth of Bacteroides and actinobacteria [51]. This may cause Bacteroidetes and actinomyces to remain at a high level and not fully recover. One limitation of our study is that the results of gut flora are descriptive and lack relevant mechanism studies. Therefore, all explanations can only be considered speculative. Therefore, further mechanistic studies, including fecal microbiome transplantation, are needed to verify the causal role of the gut microbiome in calcium-deficient osteoporosis.

In our study, a low-calcium diet was found to affect vitamin B6 metabolism, lysosome, pantothenic acid, and CoA biosynthesis, and the AMPK signaling pathway in the fecal metabolome. It also affected lysine degradation in the plasma metabolome. Bacteria-derived compounds, such as vitamins, may enter the bloodstream and directly affect bone cell activity [54]. Vitamin B6 deficiency may also be a risk factor for bone loss. Vitamin B6 is necessary for the cross-linking of lysine oxidase collagen, which plays a vital role in maintaining bone mass. Meanwhile, vitamin B6 deficiency reduces the concentration of cross-linking intermediates and disrupts collagen cross-linking in the bone [43]. The intestinal microbiome exerts a strong influence on host bone metabolism through metabolic, endocrine, and immune communication [14]. In our study, lipids accounted for a large proportion of the differential metabolites, especially SCFAs and steroid hormones. Moreover, previous studies have shown that severe vitamin B-6 deficiency alters the fatty acid profile of tissue lipids, usually accompanied by an increase in linoleic acid and a decrease in arachidonic acid. Short-term vitamin B-6 restriction reduced plasma (n-3) and (n-6) polyunsaturated fatty acids (PUFA) concentrations and tended to increase plasma (n-6): (n-3) PUFA ratios [55]. In previous studies, vitamin B6, a component of some coenzymes in the body, reduced estrogen activity and lowered estrogen levels in the body by participating in various metabolic reactions, including amino acid, protein, and estrogen metabolism. Estrogen is one of the main hormones regulating bone metabolism [56]. Sex steroid hormones in men and skeletal development and maintenance in women play a key role, and in addition to estrogen, low testosterone bioavailability may also be more likely to lead to fracture. We found that the normally-fed rats’ plasma testosterone levels were significantly higher than those in the other three groups, the decrease of testosterone low calcium for a long time, Recovery may not be achieved by calcium supplementation and the addition of chondroitin sulfate [57]. Estrogen deficiency affects bone cell-mediated remodeling, reducing bone mass and damaging bone microstructure [58]. Microbiota play a significant role in lipid homeostasis by fermenting dietary fiber for production. Bacteroides ferment soluble corn fiber into acetate or lactate, whereas Firmicutes further ferment these substrates into butyrate, thereby promoting increased calcium absorption [59]. Short-chain fatty acids, particularly propionate and butyrate, as endogenous signals [60], effectively increase the BMD by inhibiting osteoclast formation and bone resorption without directly affecting osteoblast function [61].

When calcium carbonate was administered to the low-calcium diet group, it affected the folate resistance of fecal metabolites, olfactory transduction, production and secretion of cortisol, Cushing's syndrome, and the cyclic guanosine monophosphate-protein kinase G (cGMP-PKG) signaling pathway, as well as the longevity regulation pathways of plasma metabolites—worm nicotinamide and platelet-activated ADP. Folic acid is a water-soluble vitamin that helps maintain bone density by maintaining optimal nitric oxide synthase activity in bone cells [62]. It also significantly improves bone mineral density and bone microstructure, enriches AMPK signaling pathway, and promotes the expression of lipid oxidation-related and antioxidant enzymes [63]. Folic acid is also involved in inflammation, homocysteine metabolism, and bone turnover, acting directly and indirectly on bone. Both, the olfactory transduction pathway and cGMP-PKG signaling pathway up-regulate AMP and GMP, and the non-cGMP-PKG signaling pathway plays a key role in skeletal homeostasis [64]. cGMP plays a key role in osteoblast differentiation by activating PKG, leading to local estrogen production by the stimulation of aromatase expression [65]. Estrogen antagonizes the effects of glucocorticoids through a complex interference with the function and fate of glucocorticoid receptors and physiological changes in cortisol levels may also impair bone mass [66]. Excessive cortisol production leads to decreased bone metabolism and changes in bone structure [67]. Moreover, the signs and symptoms of Cushing's syndrome caused by glucocorticoids have a range of systemic and local effects on bone and mineral metabolism that overlap with common diseases, such as metabolic syndrome, obesity, and osteoporosis [68]. The differential metabolites niacin and niacinamide in plasma-enriched lipid metabolic pathways affect skeletal homeostasis [69]. Studies have found that platelets also play a key role in bone homeostasis by regulating bone formation and bone resorption [70].

However, compared to the calcium carbonate group, under CS intervention, the difference in fecal and plasma lipid profiles of metabolites was particularly significant, and the difference in fecal metabolism was concentrated in linoleic acid metabolism. Linoleic acid may indirectly affect bone mass by stimulating calcium absorption, which has a beneficial effect on bone loss [71]. We also found that progemediated oocyte maturation, tryptophan metabolism, and purine metabolism were closely related to osteoporosis. In addition, plasma metabolic differences were enriched in glutathione metabolism. Oxidative stress is an important mediator and indicator of osteoporosis, because antioxidant enzymes such as glutathione peroxidase are more common in osteoporosis [72]. Compared with the Ca group, the cGMP-PKG signaling pathway of the CS fecal metabolites in the KEGG pathway and the antioxidant effect of cGMP in osteoblasts were mediated by PKG2. The antioxidant effect of cGMP in endothelial cells is attributed to the up-regulation of catalase and glutathione peroxidase after PKG1 transcription [73]. Biosynthesis of glutathione (GSH) is through reduction of oxidized glutathione, and GSH inhibits the nuclear factor kappa-B signaling pathway mediated by reactive oxygen species-induced osteoclast differentiation. Therefore, the GSH/NRF2-mediated antioxidant pathways are essential for the balance between osteogenesis and osteoclast formation [74]. The metabolite KEGG pathway in CS plasma is concentrated in biotin metabolism. Biotin is essential for normal cell function, growth and development. Systemic biotin deficiency can lead to developmental delay, skeletal dysplasia, immunological changes, and potential encephalopathy [75]. With CS intervention, the content of SCFAs in fecal metabolites increased compared to calcium carbonate treatment alone, and the enzyme activity contained in the intestinal microbiota digested carbohydrates and produced SCFA at a millimole concentration. The positive effects of SCFA on bone formation have been shown in animal models [76]. SCFA regulates bone homeostasis by directly inhibiting bone resorption, stimulating calcium absorption and producing immunomodulatory effects [77]. In our study, CS alleviated osteoporosis by increasing the production of SCFAs and promoting calcium absorption.

There are several mechanisms that can cause osteoporosis. But chronic low-grade inflammation has been identified as the root cause of many diseases, including osteoporosis [4]. We identified the bacteria and metabolites behind this change, especially lipid metabolism, and discussed their relevance to bone health. In fact, the innate immune receptor TLR4 has been implicated in the development of osteoporosis. Specifically, TLR4 plays a key role in the activity involved in osteoblast-mediated inflammation. In particular, the production of prostaglandin E2 is required for TLR4-mediated bone loss. Moreover, osteoblast derived nuclear factor kappa-B ligand (RANKL) determines TLR4-mediated osteoclast formation, and excessive TLR4 signal transduction implies bone destruction [46]. The SCFA we focused on reduces the host inflammatory response induced by inflammatory factors IL-1β, IL-6 and TNFα through the down-regulation of the TLR4 pathway [78]. This study provides a new strategy to elucidate the molecular mechanism of osteoporosis and to search for potential biomarkers.

## Supplementary Information

**Additional file 1: Table S1**. MRPP analysis showed significant differences in microbial community structure between group N and group C and between group C and group CS and between group CS and group Ca.

**Additional file 2: Table S2**. The differential metabolites of fecal metabolomics between the N group and the C group. The differential metabolites of fecal metabolomics between the C group and the Ca group. The differential metabolites of fecal metabolomics between the C group and the CS group. The differential metabolites of fecal metabolomics between the CS group and the Ca group.

**Additional file 3: Table S3**. The differential metabolites of plasma metabolomics between the N group and the C group. The differential metabolites of plasma metabolomics between the C group and the Ca group. The differential metabolites of plasma metabolomics between the C group and the CS group. The differential metabolites of plasma metabolomics between the CS group and the Ca group.

## Data Availability

The data that support the findings of this study are available in https://data.mendeley.com/datasets/95b49jjd25/1, https://doi.org/10.17632/95b49jjd25.1, and within the article and its supplementary materials.

## References

[1] Kanis JA (1994). The diagnosis of osteoporosis. J Bone Miner Res.

[2] Wang M (2013). Calcium-deficiency assessment and biomarker identification by an integrated urinary metabonomics analysis. BMC Med.

[3] Choi SY (2011). Effects of Sigma Anti-bonding Molecule Calcium Carbonate on bone turnover and calcium balance in ovariectomized rats. Lab Anim Res.

[4] Bott KN (2021). Trabecular and cortical bone are unaltered in response to chronic lipopolysaccharide exposure via osmotic pumps in male and female CD-1 mice. PLoS ONE.

[5] Anthamatten A, Parish A (2019). Clinical Update on Osteoporosis. J Midwifery Womens Health.

[6] Fujita T (1993). Calcium supplementation in osteoporosis. Osteoporos Int.

[7] Wallace L, Boxall M, Riddick N (2004). Influencing exercise and diet to prevent osteoporosis: lessons from three studies. Br J Community Nurs.

[8] Meiron OE (2011). Solubility and bioavailability of stabilized amorphous calcium carbonate. J Bone Miner Res.

[9] Straub DA (2007). Calcium supplementation in clinical practice: a review of forms, doses, and indications. Nutr Clin Pract.

[10] Xiao C (2021). Elemental impurities in pediatric calcium carbonate preparations-high throughput quantification and risk assessment. Front Chem.

[11] Hu Y (2021). Rhizoma drynariae total flavonoids combined with calcium carbonate ameliorates bone loss in experimentally induced Osteoporosis in rats via the regulation of Wnt3a/β-catenin pathway. J Orthop Surg Res.

[12] Ni H (2022). Meta-analysis of effects of nutritional intervention combined with calcium carbonate D3 tablets on bone mineral density, bone metabolism, and curative effect in patients with osteoporosis. Contrast Media Mol Imaging.

[13] Mikami T, Kitagawa H (2013). Biosynthesis and function of chondroitin sulfate. Biochim Biophys Acta.

[14] Mishra S, Ganguli M (2021). Functions of, and replenishment strategies for, chondroitin sulfate in the human body. Drug Discov Today.

[15] Korotkyi OH (2021). The combination of chondroitin sulfate and probiotic prevents oxidative stress in the serum of rats with experimental osteoarthritis. Minerva Biotechnol Biomol Res.

[16] Bishnoi M (2016). Chondroitin sulphate: a focus on osteoarthritis. Glycoconj J.

[17] Shen Q (2021). Fabrication of chondroitin sulfate calcium complex and its chondrocyte proliferation in vitro. Carbohydr Polym.

[18] Proal AD, Lindseth IA, Marshall TG (2017). Microbe-microbe and host-microbe interactions drive microbiome dysbiosis and inflammatory processes. Discov Med.

[19] Wang J (2017). Diversity analysis of gut microbiota in osteoporosis and osteopenia patients. PeerJ.

[20] Lin H (2020). The role of gut microbiota metabolite trimethylamine N-oxide in functional impairment of bone marrow mesenchymal stem cells in osteoporosis disease. Ann Transl Med.

[21] Zhang YW (2021). The modulatory effect and implication of gut microbiota on osteoporosis: from the perspective of "brain-gut-bone" axis. Food Funct.

[22] Korotkyi OH (2020). The gut microbiota of rats under experimental osteoarthritis and administration of chondroitin sulfate and probiotic. Mikrobiol Z.

[23] Weaver CM (2015). Diet, gut microbiome, and bone health. Curr Osteoporos Rep.

[24] Jiang YC (2020). UPLC-MS metabolomics method provides valuable insights into the effect and underlying mechanisms of Rhizoma Drynariae protecting osteoporosis. J Chromatogr B Analyt Technol Biomed Life Sci.

[25] Beger RD (2016). Metabolomics enables precision medicine: "A White Paper, Community Perspective". Metabolomics.

[26] Lv H (2016). Metabolomics and its application in the development of discovering biomarkers for osteoporosis research. Int J Mol Sci.

[27] Seely KD (2021). The human gut microbiota: a key mediator of osteoporosis and osteogenesis. Int J Mol Sci.

[28] Caporaso JG (2010). QIIME allows analysis of high-throughput community sequencing data. Nat Methods.

[29] Bokulich NA (2013). Quality-filtering vastly improves diversity estimates from Illumina amplicon sequencing. Nat Methods.

[30] Edgar RC (2011). UCHIME improves sensitivity and speed of chimera detection. Bioinformatics.

[31] Haas BJ (2011). Chimeric 16S rRNA sequence formation and detection in Sanger and 454-pyrosequenced PCR amplicons. Genome Res.

[32] Quast, C., et al., The SILVA ribosomal RNA gene database project: improved data processing and web-based tools. Nucleic Acids Res, 2013. **41**(Database issue): D590–6. 10.1093/nar/gks1219 PMC3531112 23193283

[33] Luo P (2015). Multiple reaction monitoring-ion pair finder: a systematic approach to transform nontargeted mode to pseudotargeted mode for metabolomics study based on liquid chromatography-mass spectrometry. Anal Chem.

[34] He J (2020). Gut microbiota and metabolite alterations associated with reduced bone mineral density or bone metabolic indexes in postmenopausal osteoporosis. Aging (Albany NY).

[35] Cheng M (2021). Gut microbiota is involved in alcohol-induced osteoporosis in young and old rats through immune regulation. Front Cell Infect Microbiol.

[36] Yatsonsky Ii D (2019). Linkage of microbiota and osteoporosis: a mini literature review. World J Orthop.

[37] Wei M (2021). High-throughput absolute quantification sequencing revealed osteoporosis-related gut microbiota alterations in han chinese elderly. Front Cell Infect Microbiol.

[38] Chiu YH (2014). Lactobacillus casei MYL01 modulates the proinflammatory state induced by ethanol in an in vitro model. J Dairy Sci.

[39] McCabe LR (2019). Exercise prevents high fat diet-induced bone loss, marrow adiposity and dysbiosis in male mice. Bone.

[40] Di DS (2021). Integrative analysis of LGR5/6 gene variants, gut microbiota composition and osteoporosis risk in elderly population. Front Microbiol.

[41] Ma S (2021). Fecal microbiota transplantation mitigates bone loss by improving gut microbiome composition and gut barrier function in aged rats. PeerJ.

[42] Rettedal EA (2021). The gut microbiome is altered in postmenopausal women with osteoporosis and osteopenia. JBMR Plus.

[43] Ling CW (2021). The association of gut microbiota with osteoporosis is mediated by amino acid metabolism: multiomics in a large cohort. J Clin Endocrinol Metab.

[44] Li C (2019). Gut microbiota composition and bone mineral loss-epidemiologic evidence from individuals in Wuhan. China Osteoporos Int.

[45] Li M (2018). Pro- and anti-inflammatory effects of short chain fatty acids on immune and endothelial cells. Eur J Pharmacol.

[46] Alonso-Pérez A (2018). Role of toll-like receptor 4 on osteoblast metabolism and function. Front Physiol.

[47] Sisignano M, Fischer MJM, Geisslinger G (2021). Proton-sensing GPCRs in health and disease. Cells.

[48] Collins, F.L., et al. The potential of probiotics as a therapy for osteoporosis. Microbiol Spectr, 2017. 5(4). 10.1128/microbiolspec.bad-0015-2016 PMC5710820 28840819

[49] Shang Q (2016). Degradation of chondroitin sulfate by the gut microbiota of Chinese individuals. Int J Biol Macromol.

[50] Ozaki D (2021). Association between gut microbiota, bone metabolism, and fracture risk in postmenopausal Japanese women. Osteoporos Int.

[51] Chaplin A (2016). Calcium supplementation modulates gut microbiota in a prebiotic manner in dietary obese mice. Mol Nutr Food Res.

[52] Shang Q (2016). Structural modulation of gut microbiota by chondroitin sulfate and its oligosaccharide. Int J Biol Macromol.

[53] Shmagel A (2019). The effects of glucosamine and chondroitin sulfate on gut microbial composition: a systematic review of evidence from animal and human studies. Nutrients.

[54] Rizzoli R, Biver E (2020). Are probiotics the new calcium and vitamin D for bone health?. Curr Osteoporos Rep.

[55] Zhao M (2012). Marginal vitamin B-6 deficiency decreases plasma (n-3) and (n-6) PUFA concentrations in healthy men and women. J Nutr.

[56] Mastrangelo M, Cesario S (2019). Update on the treatment of vitamin B6 dependent epilepsies. Expert Rev Neurother.

[57] Cauley JA (2015). Estrogen and bone health in men and women. Steroids.

[58] Li Z (2021). Vitamin B6 as a novel risk biomarker of fractured ankles. Medicine (Baltimore).

[59] Whisner CM (2016). Soluble corn fiber increases calcium absorption associated with shifts in the gut microbiome: a randomized dose-response trial in free-living pubertal females. J Nutr.

[60] Hills RD (2019). Gut microbiome: profound implications for diet and disease. Nutrients.

[61] Lucas S (2018). Short-chain fatty acids regulate systemic bone mass and protect from pathological bone loss. Nat Commun.

[62] Harre U (2015). Glycosylation of immunoglobulin G determines osteoclast differentiation and bone loss. Nat Commun.

[63] He H (2021). Folic acid attenuates high-fat diet-induced osteoporosis through the AMPK signaling pathway. Front Cell Dev Biol.

[64] Kim SM (2021). The NO-cGMP-PKG pathway in skeletal remodeling. Ann N Y Acad Sci.

[65] Wisanwattana W (2021). Inhibition of phosphodiesterase 5 promotes the aromatase-mediated estrogen biosynthesis in osteoblastic cells by activation of cGMP/PKG/SHP2 pathway. Front Endocrinol (Lausanne).

[66] Osella G (2012). Cortisol secretion, bone health, and bone loss: a cross-sectional and prospective study in normal non-osteoporotic women in the early postmenopausal period. Eur J Endocrinol.

[67] Al-Rawaf HA, Alghadir AH, Gabr SA (2021). Circulating MicroRNA expression, vitamin D, and hypercortisolism as predictors of osteoporosis in elderly postmenopausal women. Dis Markers.

[68] Rubinstein G (2020). Time to diagnosis in cushing's syndrome: a meta-analysis based on 5367 patients. J Clin Endocrinol Metab.

[69] Li Y (2011). Nicotinamide phosphoribosyltransferase (Nampt) affects the lineage fate determination of mesenchymal stem cells: a possible cause for reduced osteogenesis and increased adipogenesis in older individuals. J Bone Miner Res.

[70] Salamanna F (2020). Platelet features and derivatives in osteoporosis: a rational and systematic review on the best evidence. Int J Mol Sci.

[71] Kelly O, Cashman KD (2004). The effect of conjugated linoleic acid on calcium absorption and bone metabolism and composition in adult ovariectomised rats. Prostaglandins Leukot Essent Fatty Acids.

[72] Zhao F (2021). Correlation of oxidative stress-related biomarkers with postmenopausal osteoporosis: a systematic review and meta-analysis. Arch Osteoporos.

[73] Kalyanaraman H (2018). Protein kinase G activation reverses oxidative stress and restores osteoblast function and bone formation in male mice with type 1 diabetes. Diabetes.

[74] Yang K (2022). Three classes of antioxidant defense systems and the development of postmenopausal osteoporosis. Front Physiol.

[75] Subramanian VS (2017). Mutations in SLC5A6 associated with brain, immune, bone, and intestinal dysfunction in a young child. Hum Genet.

[76] Duscha A (2022). Propionic acid beneficially modifies osteoporosis biomarkers in patients with multiple sclerosis. Ther Adv Neurol Disord.

[77] Li J (2021). The role of gut microbiota in bone homeostasis. Bone Joint Res.

[78] Sam QH (2021). The divergent immunomodulatory effects of short chain fatty acids and medium chain fatty acids. Int J Mol Sci.

